# Projected impacts of future climate change, ocean acidification, and management on the US Atlantic sea scallop (*Placopecten magellanicus*) fishery

**DOI:** 10.1371/journal.pone.0203536

**Published:** 2018-09-21

**Authors:** Jennie E. Rheuban, Scott C. Doney, Sarah R. Cooley, Deborah R. Hart

**Affiliations:** 1 Department of Marine Chemistry and Geochemistry, Woods Hole Oceanographic Institution, Woods Hole, Massachusetts, United States of America; 2 Department of Environmental Sciences, University of Virginia, Charlottesville, Virginia, United States of America; 3 The Ocean Conservancy, Washington, DC, United States of America; 4 Northeast Fisheries Science Center, Woods Hole, Massachusetts, United States of America; University of Hong Kong, HONG KONG

## Abstract

Ocean acidification has the potential to significantly impact both aquaculture and wild-caught mollusk fisheries around the world. In this work, we build upon a previously published integrated assessment model of the US Atlantic Sea Scallop (*Placopecten magellanicus)* fishery to determine the possible future of the fishery under a suite of climate, economic, biological, and management scenarios. We developed a 4x4x4x4 hypercube scenario framework that resulted in 256 possible combinations of future scenarios. The study highlights the potential impacts of ocean acidification and management for a subset of future climate scenarios, with a high CO_2_ emissions case (RCP8.5) and lower CO_2_ emissions and climate mitigation case (RCP4.5). Under RCP4.5 and the highest impact and management scenario, ocean acidification has the potential to reduce sea scallop biomass by approximately 13% by the end of century; however, the lesser impact scenarios cause very little change. Under RCP8.5, sea scallop biomass may decline by more than 50% by the end of century, leading to subsequent declines in industry landings and revenue. Management-set catch limits improve the outcomes of the fishery under both climate scenarios, and the addition of a 10% area closure increases future biomass by more than 25% under the highest ocean acidification impacts. However, increased management still does not stop the projected long-term decline of the fishery under ocean acidification scenarios. Given our incomplete understanding of acidification impacts on *P*. *magellanicus*, these declines, along with the high value of the industry, suggest population-level effects of acidification should be a clear research priority. Projections described in this manuscript illustrate both the potential impacts of ocean acidification under a business-as-usual and a moderately strong climate-policy scenario. We also illustrate the importance of fisheries management targets in improving the long-term outcome of the *P*. *magellanicus* fishery under potential global change.

## 1 Introduction

Ocean acidification, the process where increased atmospheric carbon dioxide (CO_2_) concentration reduces ocean pH and calcium carbonate saturation state [1], has already been implicated in negative impacts on the aquaculture industry on the US West Coast [2]. Laboratory studies for a number of other economically important marine species suggest ocean acidification has the potential to significantly impact fisheries landings and revenues regionally and around the world [3–6]. Marine mollusks appear to be particularly sensitive to ocean acidification due to the sensitivity of the calcification process to seawater chemistry during shell building [6–8]. Mollusks make up approximately 22 and 15% of the wild-caught fisheries yield globally and in the US, respectively [9]; therefore, understanding sensitivities of wild-caught fisheries to ocean acidification is an important step in the long-term sustainable management of these species.

Marine mollusks, specifically bivalves, are impacted in many ways by acidification. Their often complex, multi-stage life-cycle leads to potential bottlenecks where the chemical environment is particularly important, such as during metamorphoses from various larval stages prior to settlement [10]. Many species have shown increased larval mortality due to acidification, and larvae that survive often have deformed shell structures that suggest potential reductions in fitness during juvenile or adult stages [10–12]. Experimental manipulations of juvenile and adult bivalves suggest reduced growth and calcification rates under acidified conditions [7, 8, 13]. Further, shellfish predator-prey interactions are hypothesized to be influenced by acidification, due to decreased shell thickness and strength [14], reduced escape performance [15], and decreased behavioral responses to predators [14].

The US Atlantic sea scallop (*Placopecten magellanicus*) is an ideal test species to apply integrated assessment modeling to project the impacts of global change on a wild-caught fishery. The *P*. *magellanicus* fishery is one of the most valuable wild-caught fisheries in the US, regularly worth roughly $500 million (USD) in ex-vessel revenue [16]. This fishery was severely overfished in the early to mid-1990s, but was rapidly rebuilt by the early 2000s using a combination of fishing effort reductions, gear restrictions, and rotational as well as long-term fishery closures [17]. Because the *P*. *magellanicus* fishery is currently sustainable, stakeholders and managers have the opportunity to consider the impacts of long-term environmental change on the fishery stock, yield, and economic benefits.

In order to maintain a sustainable fishery into the future, it is important that all stakeholders involved in both the management and exploitation of the fishery understand possible futures of the *P*. *magellanicus* fishery and the potential impacts of decision-making. In this work, we expand on the model described in Cooley et al. [13] by incorporating different future scenarios to inform planning for long-term change. Moreover, new elements have been added to the model to include the economic development associated with different growth and emissions scenarios (the RCP pathways); economic development was previously held constant in Cooley et al. [13]. We designed a decision-support framework that explores multiple combinations of potential future scenarios to provide an ensemble of model output varying major influences on the fishery as a whole. In this manuscript, we describe a subset of those scenarios to address the following questions: how might the future trajectory of the *P*. *magellanicus* fishery change under future climate warming and ocean acidification? How do differing management regimes influence the long-term outcomes of the *P*. *magellanicus* fishery? In this work, we designed future scenarios that incorporate potential acidification impacts, future climate scenarios that affect economic development, fisheries management, and changing fuel costs (described in more detail below).

## 2 Methods

**Fig 1:**
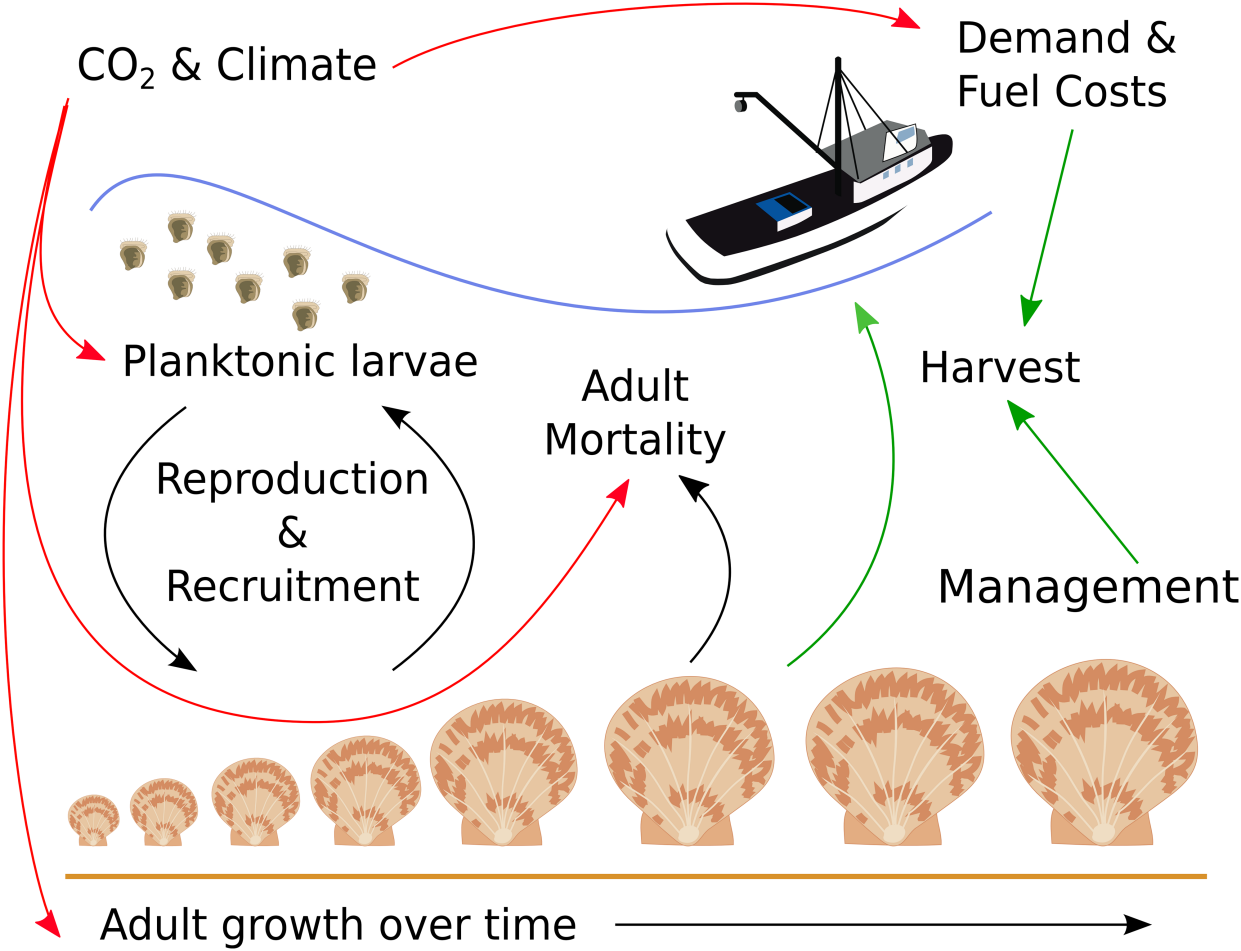
Conceptual illustration of the integrated assessment model. Red arrows indicate connections between submodels, black arrows indicate population submodel, green arrows indicate decision-making and socio-economic submodel. Illustrations courtesy of the Integration and Application Network, University of Maryland Center for Environmental Science (ian/umces/edu/symbols/).

Cooley et al. [13] describe an integrated assessment model (IAM) for the *P*. *magellanicus* fishery. This model combines elements of fisheries management models that include matrix population models and socio-economic models with reduced-form biogeochemical modeling to project the future fishery response to changes in environmental and economic conditions. The IAM incorporates ocean acidification and warming into fisheries projections through altered growth rates and larval survival. Based on economic viability and stock biomass, the IAM forecasts fisheries yield and economic benefits under future conditions [13]. We briefly summarize the model design below. A conceptual illustration of the IAM is given in Fig 1.

### 2.1 Integrated assessment model

The scallop population submodel is a size-based matrix population model that was simplified from the sea scallop fisheries management model, the Scallop Area Management Simulator (SAMS). The SAMS model splits the scallop population into a number of distinct geographic units with individual populations and growth rates based on historical survey data. The reduced population submodel used in the IAM described in Cooley et al. [13] separates the sea scallop population into two geographic units, Georges Bank and the Mid-Atlantic Bight, and tracks each population with unique population parameters. The population submodel includes terms for recruitment, growth, maximum size, and natural, incidental, discard, and fishing mortalities, growth rates, maximum sizes, and recruitment.

The biogeochemical submodel is a two-box ocean model (surface and bottom waters) with separate sub-modules for Georges Bank and Mid-Atlantic Bight regions. Carbonate chemistry is explicitly modeled including terms for air-sea gas exchange, primary production, and calcium carbonate production in the surface box, and biological export and organic matter remineralization in the bottom water box. The two-box model also includes terms for temperature and salinity to determine seasonal changes in stratification and mixing, and the biogeochemical model parameters were trained with data from the 2000–2010 period. The biogeochemical model in the IAM is linked to the population model through temperature and calcium carbonate mineral saturation state impacts on adult growth and larval mortality. The biogeochemical model is linked to the socio-economic model through the future climate scenarios, from the IPCC Representative Concentration Pathways (RCPs), which set future atmospheric CO_2_ concentration.

The economic submodel is based on economic modeling of the sea scallop fishery by the New England Fisheries Management Council [18]. The submodel uses price and cost models that include terms for landings, per-capita disposable income (an indication of economic development), scallop imports and exports, fuel costs, days at sea, and number of crew members. From yearly estimated price and costs, the economic model determines the optimal number of days at sea to fish based on a rules analysis maximizing net profit and adhering to management set maximum allowable biological catch (ABC) that is determined as the catch associated with a precautionary fishing mortality (F_ABC_) that is a slight reduction in the fishing mortality at maximum sustainable yield (F_MSY_). The economic submodel is linked to the scallop population model through the management set limits on catch and the scallop pricing analysis, and linked to the biogeochemical model through the RCP scenarios.

### 2.2 Updates to the IAM and future projections

To explore the future of the US Atlantic sea scallop industry under climate scenarios over the next century, we identified four major areas of interest: 1) potential ocean acidification impacts, 2) future climate scenarios that incorporate economic development, 3) fishery management scenarios, and 4) future fuel costs. Below, we describe the major features of each scenario.

#### 2.2.1 Ocean acidification impacts

**Fig 2:**
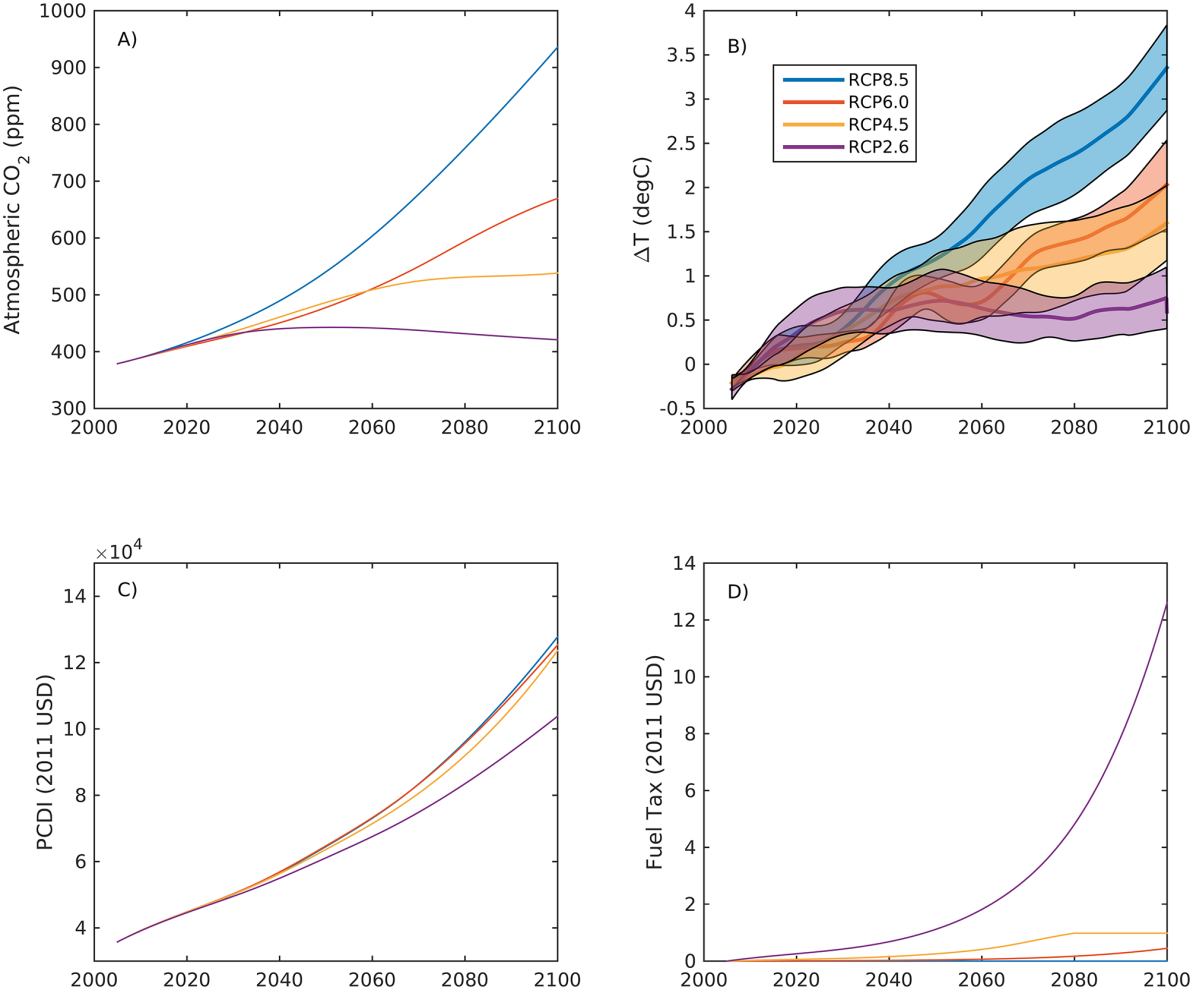
External drivers of integrated assessment model. A) Atmospheric CO_2_ from the Representative Concentration Pathways (RCPs), B) change in sea surface temperature from 2000–2006 (mean ± SD) from 10 global earth system models in the 10x10 degree region containing the Mid-Atlantic Bight and Georges Bank scallop populations, C) US per-capita disposable income trajectories corrected to 2011 USD, and D) carbon tax converted to potential diesel fuel tax associated with each RCP.

Climate and biogeochemical scenarios were developed from the IPCC AR5 RCP scenarios. Major biogeochemical influences include atmospheric CO_2_ and oceanic temperatures. Atmospheric CO_2_ trajectories were used from the RCPs [19] (Fig 2A), and temperature trends were obtained from the 10 global earth system models used in Bopp et al. [20] from the CMIP5 database. The projected SST fields from RCP2.6, 4.5, 6, and 8.5 were interpolated to a 1°x1° grid consistent across all models. We quantified regional trends in area-weighted mean monthly SST fields from a 10° x 10° area containing the Georges Bank and Mid-Atlantic Bight regions in the Northwest Atlantic. We quantified anomalies from the mean temperatures of the first five years of future projections (2006–2010), and applied stochastic temperature trajectories from the projection distribution derived from the model ensemble to the biogeochemical submodel (Fig 2B, SST).

Four sets of increasing biological impacts were simulated representing none, low, medium and high impacts. “No” impact is defined as maintaining constant growth rates and recruitment throughout the simulation independent of temperature and CO_2_. Although no studies to date regarding ocean acidification response have been published on *P*. *magellanicus*, from other bivalve species, we expect that adult growth or calcification rates and larval survival may be negatively impacted by rising CO_2_ [13]. “Low” impacts of ocean acidification are defined as reductions in larval survival with carbonate mineral saturation state alone, while “medium” impacts are both reductions in larval survival with saturation state and decreased growth and calcification rates, as described by Cooley et al. [13] as a function of saturation anomaly.

The “high” impact case is defined by other possible impact pathways beyond just growth and calcification. Recent work on mollusk species suggests that predator-prey interactions such as behavior, cue detection, or shell thickness may be altered as a result of ocean acidification [14]. Mollusk shells are the first line of defense for many species, and if shell thickness is compromised, scallops may be more susceptible to predation [14]. Early studies from *Pecten maximus*, a similar species of scallop found in the Northeast Atlantic, suggests that swimming behavior may be compromised due to ocean acidification, reducing the escape performance of scallops under future climate scenarios [15, 21]. The majority of predation on juvenile *P*. *magellanicus* (20-90mm shell height) is due to *Cancer spp*. crabs; these scallops generally can escape sea stars (e.g., *Asterias* spp.) by swimming. Scallops larger than 70-90mm are currently invulnerable to *Cancer* predation due to their thick shells [22]. We hypothesize that ocean acidification may reduce both shell strength and swimming ability, thus reducing the scallops’ defenses against both type of predators. Because there are no empirical relationships to estimate these effects, in the “high” impact case we increase natural mortality of small scallops (< 90mm) proportionally with the change in saturation state.

#### 2.2.2 Economic development

The major drivers of the socioeconomics of the industry are economic development, specifically per capita disposable income (PCDI, which impacts scallop pricing and demand) and fuel costs (which impacts industry operating costs and crew incomes). To develop trajectories for future socioeconomic scenarios, we used the output from the GCAM integrated assessment model which developed the RCP4.5 scenario [23–25]. Each official RCP scenario has been generated using a different integrated assessment model with separate underlying drivers and assumptions. In order to maintain consistency between model assumptions for the future scenarios, we used projections of economic development and carbon taxes from GCAM’s estimates of the other RCP scenarios. We converted US GDP and population projections to PCDI by correcting GDP per capita data to PCDI through a derived correlation using historical data from US Bureau of Economic Analysis of US population (pop), GDP, and PCDI data corrected to 2011 USD (Eq 1):

$${PCDI}_{2011}=A*(\frac{{GDP}_{2011}}{pop})-B,$$ (1)

where PCDI_2011_ is the historical per capita disposable income corrected to 2011 USD, GDP_2011_ is the historical US GDP corrected to 2011 USD, and pop is the historical population. A and B are constants derived as 0.8025 ± 0.0286 and 2383 ± 1089 (r^2^ = 0.984, p < 0.0001), respectively. Future PCDI, corrected to 2011 USD, is expected to increase by a factor of 2.5–3 by the end of century under all four climate scenarios analyzed in this study (Fig 2C). There is considerable uncertainty in projections of energy use, emissions scenarios, and economic development that has begun to be recently explored in greater detail using the future Shared Socioeconomic Pathways (SSPs) [26]. Given that our temperature and atmospheric CO_2_ projections were based upon the RCP scenarios, we chose to the use the RCP projections of GDP and population to generate our PCDI projection. The impacts of different economic development narratives could be explored in more detail using the new SSPs but would be beyond the scope of this study.

#### 2.2.3 Management

To test the impacts of management of the sea scallop industry on future scenarios, we test four different management cases:

1. No catch limits: Although not realistic, we allow the current limited access scallop fleet (~320 vessels) to choose scallop landings based on economic viability of fishing through the choice of days at sea from 0 to 365. This removes the catch and effort limits from the economic decision-making rules described in Cooley et al. [13], while maintaining the scallop fleet as a limited access fishery with its current gear regulations, crew limits and selectivity.
2. Low management: Regulations are set at those described in Cooley et al. [13]. Management sets annual catch limits by setting fishing mortality to F_ABC_, and tracking catch [13, 27, 28]. This reduction from F_MSY_ accounts for scientific uncertainty in biological reference points. This management scenario assumes that fisheries reference points (e.g. F_MSY_) remain constant through time.
3. Medium management: This assumes the rules from “low management,” and additionally quantifies fisheries reference points every 5 years based on updated growth rates and natural mortality from changing local temperature and carbonate mineral saturation state. This scenario changes F_MSY_ over time based on growth and mortality rates using the stochastic yield-per-recruit model described in Hart [29] and is used by NMFS during their regular stock assessment workshops [27, 28] to reevaluate fisheries reference points.
4. High management: This scenario assumes the rules from “medium” management scenarios, and additionally adds in a closure that represents a permanently closed area to fishing. Beginning in 2012, we assume 10% of the biomass of each Georges Bank and the Mid-Atlantic Bight populations are placed in permanently closed areas and track the two sub-populations separately over time. This biomass adds to the total supply of recruits, but is not available for fishing, so that fishing mortality and the associated incidental and discard mortalities for this subpopulation are zero. We assume 10% of the total recruitment occurs in closed areas each year. The closed area biomass is then factored into both the stochastic yield model to quantify F_MSY_, and the forward projecting model. This scenario is most similar to the current management scheme, which includes permanently closed regions (parts of Closed Area II) on Georges Bank that represents approximately 10% of the biomass.

#### 2.2.4 Fuel costs

Fuel costs can be up to 80% of scallop boat operating costs [18]. In a scenario incorporating climate policies, future fossil fuel prices may be strongly influenced by possible carbon taxation levels. The US EIA [30] develops scenarios to predict future fuel costs under various economic and policy scenarios. The reference case assumes current policies and economic development, which projects that the nominal diesel fuel price per gallon will increase at a rate of 0.7%/yr. To estimate the impact of climate policy on fuel costs, the EIA assumes climate policies where $1 per ton carbon tax is equivalent to $0.01 per gallon diesel fuel tax (Fig 2D). In our scenario framework, we apply four different fuel price growth rates (1.4%, 0.7%, 0.35%, 0%) combined with carbon taxes from the GCAM model output which are required to reach the specific RCP pathway such that fuel costs are calculated as:

$${Fuel}_{i,j,t}={Fuel}_{0}e^{r_{i}t}+{CarbonTax}_{j,t}/100,$$ (2)

where *Fuel*_0_ is the initial fuel cost in 2012, *r*_i_ is the fuel price growth rate of the scenario *i*, and *CarbonTax*_j,t_ is the carbon tax estimated using GCAM for climate scenario *j* in year *t*.

The set of scenarios described above generate a 4x4x4x4 hypercube of 256 possible combinations of impacts, ranging from no change of any drivers, to a high degree of climate impacts, management, and economic change (Table 1). For each future scenario, the full IAM is run with stochastically varying recruitment parameters drawn from distributions determined by the underlying data (see [13]) and stochastically varying future temperatures determined from the projections shown in Fig 2. All figures shown summarize an ensemble of 100 model runs for each scenario, and the results for each scenario are presented as the mean ± one standard deviation from the ensemble. The stochastic model runs for all scenarios yielded 25,600 individual simulations.

**Table 1:** 4x4x4x4 scenario hypercube framework. Climate scenarios incorporate changes in fuel costs associated with carbon taxation as well as economic development used in the GCAM projections. Total fuel price is calculated as the sum of the base price, determined from the growth rate, and the carbon tax. Ocean (OA) acidification impacts include either no impact, or combinations of increasing larval mortality (Larvae), reduced growth rates (Growth), and increased mortality of small scallops due to predation (Predation). Management impacts include either no set catch limits, or combinations of maximum allowable biological catch (ABC), varying reference points due to changing growth rates (F_MSY_), and closed areas (10% closure).

**Scenario Impacts**	**Increasing degree of impact →**			
**Base fuel growth rate (%)**	0	0.35	0.7	1.4
**Climate (CO**_**2**_**, Temperature)**	**RCP2.6**	**RCP4.5**	**RCP6.0**	**RCP8.5**
PCDI_2095_ (2011 USD)	98400	114500	117400	119200
Fuel Tax_2095_ ($/ton Carbon/100)	10.004	0.982	0.356	0.00
Fuel price	Base fuel price + Fuel tax			
**OA Impacts**	**None**	**Low**	**Medium**	**High**
	None	Larvae	Larvae + Growth	Larvae + Growth + Predation
**Management**	**None**	**Low**	**Medium**	**High**
	No catch limit	ABC	ABC + F_MSY_	ABC + F_MSY_ + Area Closures

Some of our scenarios overlap in design with the results presented in Cooley et al. [13]. Similar scenarios include forcings of RCP8.5, ABC only management, larval mortality and growth rate impacts, and no fuel cost increases. However, these overlapping scenarios differ substantially in underlying socio-economic conditions because this analysis includes the economic development associated with the RCPs that were held constant in Cooley et al. [13]. For example, all four RCPs project increasing GDP, population, and PCDI (Fig 2), which affect the national demand for and price of scallops.

## 3 Results and discussion

This study analyzes the future of the *P*. *magellanicus* fishery under the 4x4x4x4 hypercube that generates 256 possible combinations of impacts, ranging from no change of any drivers, to a high degree of climate impacts, management, and economic change, including both increases in fuel costs and GDP as well as population growth (Table 1). Of the full database, we focus this manuscript on describing the patterns in the impacts of management and ocean acidification scenarios, for RCP8.5 and RCP4.5, a business-as-usual and moderate-strength climate policy scenario, respectively.

### 3.1 Impacts of ocean acidification and climate scenario

Model projections show that the degree of ocean acidification impact, climate scenario, and level of management largely determine the future pathway of the *P*. *magellanicus* fishery. Fuel cost, even when including both high prices and high carbon taxes, had minimal impact, because at current biomass and scallop price per pound, decisions on how many days to fish are not strongly influenced by fuel prices. Under scenarios where biomass declines to a small fraction of current levels and fuel costs increase dramatically (e.g. no set catch limits, high ocean acidification impacts, high fuel price growth rates, and high carbon taxes), increasing fuel costs do play a role in reducing the number of days fished.

**Fig 3:**
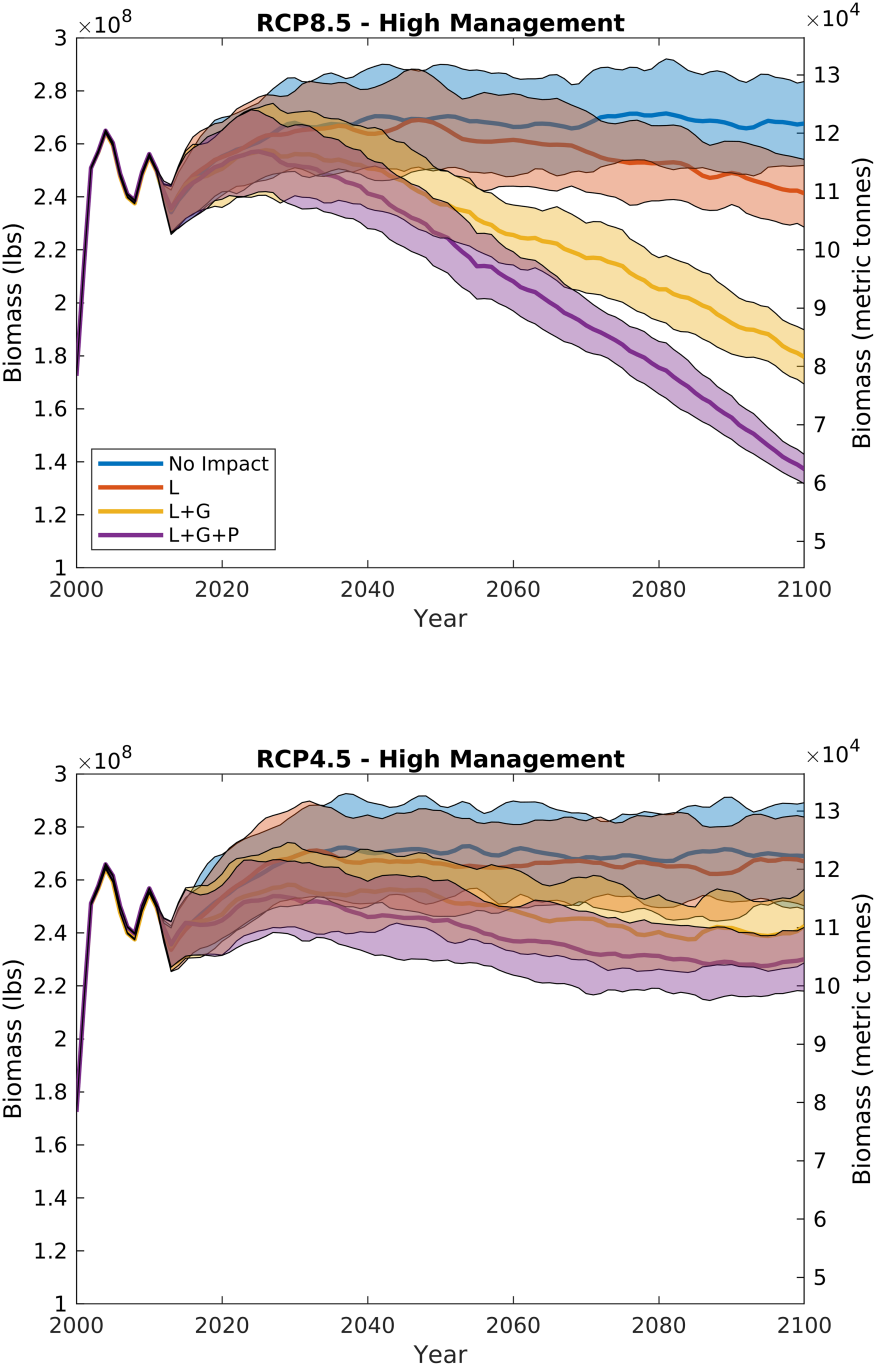
Scallop whole stock biomass from RCP8.5 (top panel) and RCP4.5 (bottom panel) under the highest management strategy (catch limits, variable reference points, and 10% area closure) with varying additive ocean acidification impacts: Decreased larval survival (L), reduced growth rates (L+G), and increased predation on small scallops (L+G+P). Bold line illustrates the mean, and shaded area is the 95% confidence interval for 100 model runs with stochastic recruitment. The 2000–2012 period is hindcast with observed recruitment.

**Fig 4:**
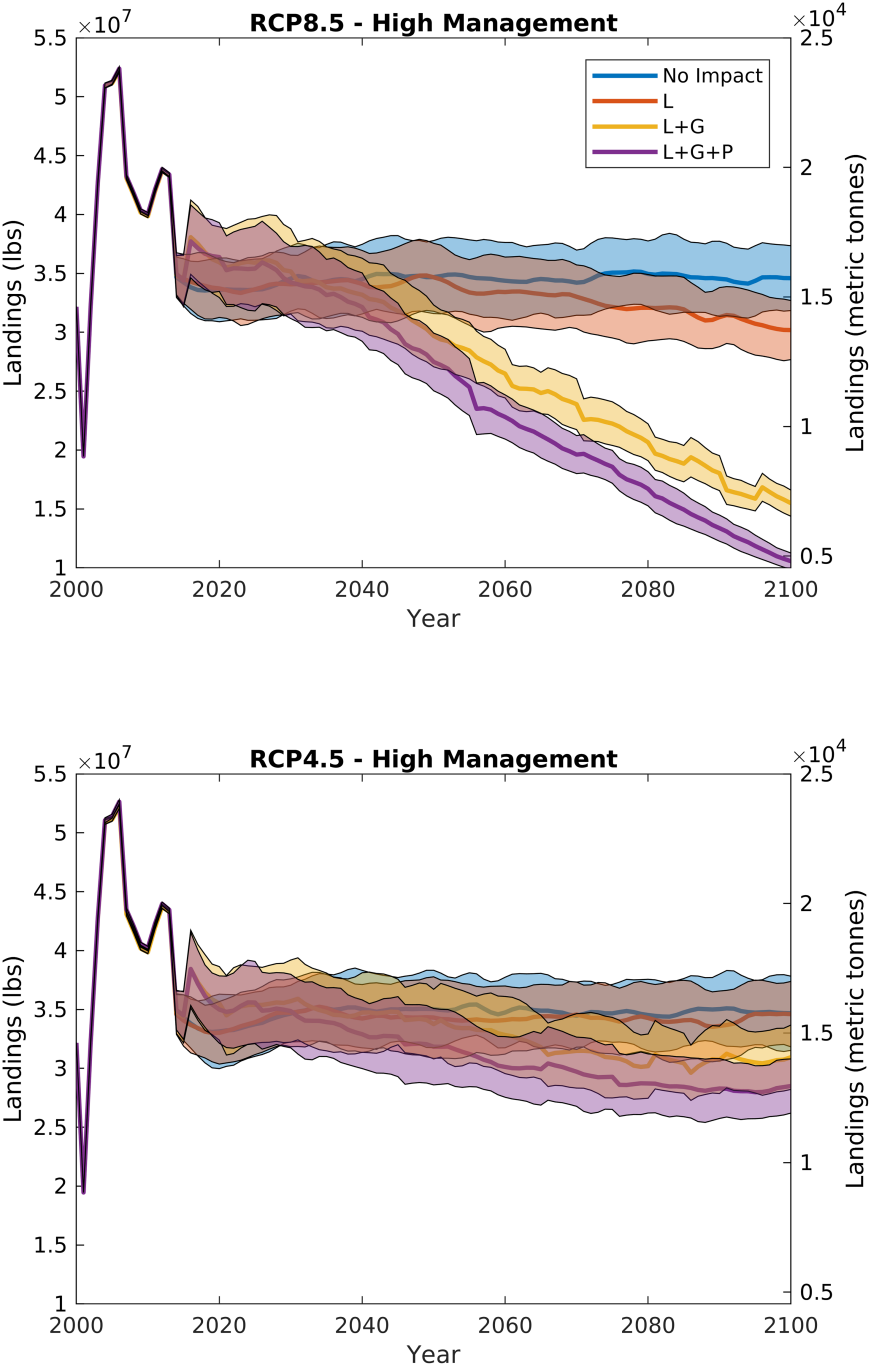
Scallop fishery landings from RCP8.5 (top panel) and RCP4.5 (bottom panel) under the highest management strategy (catch limits, variable reference points, and 10% area closure) with varying ocean acidification impacts: Decreased larval survival (L), reduced growth rates (L+G), and increased predation on small scallops (L+G+P). Bold line illustrates the mean, and shaded area is the 95% confidence interval for 100 model runs with stochastic recruitment. The 2000–2012 period is hindcast with observed recruitment.

In control experiments where management remains similar to what exists today, i.e., catch limits, regularly updated fisheries management targets, and area closures (e.g. similar to our high management scenario), and there are no impacts of ocean acidification and warming, biomass is likely to be stable through the end of century (blue curves in Fig 3). Our model does predict a slight increase in biomass that stabilizes around 2050 (Fig 3), and stable landings (Fig 4) for the no OA impact cases for both the RCP8.5 and RCP4.5 future climate scenarios due to the addition of a 10% area closure beginning in 2012 under the highest management scenario. With no climate or acidification impacts, a stable future of the scallop fishery is not surprising, given that current fisheries management targets under the Magnuson-Stevens Fishery Conservation and Management Act are designed such that harvest is maintained at a sustainable level into the future assuming no changes to biological parameters and fishery reference points [31].

When ocean acidification and warming impacts are added to the model, the future trajectory of the fishery changes, with declines projected in both stock and landings. Under RCP4.5 and high management, increasing ocean acidification impact has a small effect on the future of the fishery (Figs 3 and 4, bottom panels); because the RCP4.5 climate policy scenario limits atmospheric CO_2_ increases to less than 550 ppm (Fig 2A), the resulting small declines in carbonate mineral saturation state from present-day conditions do not translate to major fishery impacts. However, under RCP8.5 and high management, because increases in atmospheric CO_2_ (>900 ppm) and temperature (>+3 deg. C) (Fig 2A and 2B) lead to projected declines in both surface and bottom water carbonate mineral saturation state, increasing ocean acidification impact dramatically changes the future of the fishery, with whole stock biomass and landings declines ranging from 10 to 50% of the end-of-century estimates with no ocean acidification impacts (Figs 3 and 4, top panels).

Larval mortality alone does not begin impacting scallop population dynamics until mid-century, when saturation state is low enough (~1.) that it begins influencing larval supply. Historically, the scallop subpopulation on Georges Bank has not shown a strong stock-recruit relationship [17, 28]. The lack of a clear relationship is likely due to larval retention and saturation within the gyre over Georges Bank [17] rather than a function of larval supply. The IAM in its current form assumes that increases in larval mortality with ocean acidification [7, 8, 12] manifest in the population as a decline in recruitment [13], but this impact only becomes prominent on the more linear portion of the stock-recruit function. For example, because the Georges Bank stock-recruit function approaches its asymptote at a low biomass, under present day populations, a 50% increase in larval mortality on Georges Bank may only lead to a ~1.6% decline in recruitment [13]. The stock-recruit curve for the Mid-Atlantic subpopulation saturates much more slowly [28, 29], so increasing larval mortality with ocean acidification has a greater relative impact on this subpopulation. At present, the Mid-Atlantic subpopulation is roughly 40–60% of the yearly combined Mid-Atlantic and Georges Bank total stock biomass, depending on survey methodology [28]. Because recruitment impacts affect the subpopulations at different rates, declines due to larval mortality alone may change the spatial distribution towards larger relative populations on Georges Bank. However, declines due to ocean acidification on the whole stock are still not distinguishable from the interannual variability expected among different year-classes until the latter half of the century for the larval case L for RCP8.5 (red data in Fig 3, top panel). With the addition of growth rate impacts and increased mortality of small scallops due to predation, the scallop resource is projected to decline faster under RCP8.5, leading to ~10%, and ~30%, reductions in biomass and landings, respectively by 2050, and ~50%, and ~70% by end of century (Figs 3 and 4).

**Fig 5:**
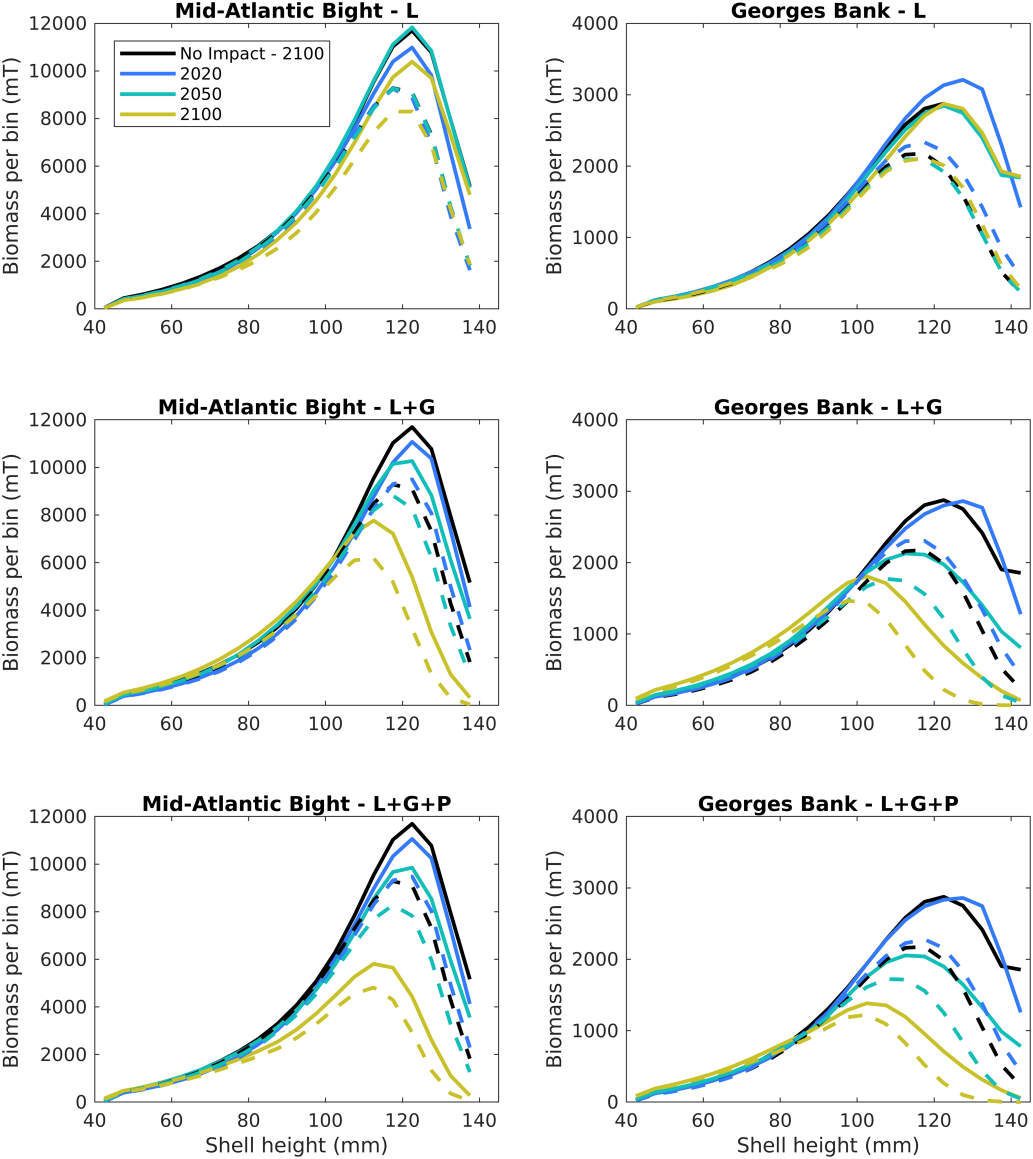
Scallop biomass by size class from the Mid-Atlantic Bight (left panels) and Georges Bank (right panels) populations versus ocean acidification impact (rows). Upper panels show larval impacts only, middle panels show larval and growth impacts, and bottom panels show larval, growth, and increased predation impacts. Dashed lines illustrate catch limit only management scenarios and solid lines illustrate the highest level of management (catch limits, varying reference points, and 10% area closures). Black lines indicate reference simulations with no ocean acidification impacts.

In addition to declining overall biomass, changes in scallop population size distribution are evident with increased ocean acidification impacts. Fig 5 illustrates the impacts of both ocean acidification on different life stages (described below, different panels) and management (described in section 3.2, solid vs. dashed lines) over time (colors) on scallop biomass size distributions. The solid lines for each panel of Fig 4 map to the projections in Fig 3 for RCP8.5. Scallop population distributions reflect declines in both overall biomass over time (Fig 3, colors in Fig 5), and wholesale shifts in population towards smaller scallops compared to scenarios with no ocean acidification or warming impacts (Fig 5, colors vs. black). This shift towards a smaller sized population is a direct result of the positive feedback from the combined reduction in growth rates and increases in mortality of small scallops plus targeted harvests of the largest sized scallops.

A population composed of smaller scallops has significant implications for the overall fishery. Under the highest ocean acidification impact—larval, growth, and juvenile mortality (L+G+P)–population distribution changes from shell height modes in 2020 at ~125mm and ~120mm for the Georges Bank and Mid-Atlantic populations, respectively, to ~110mm and 100mm by end of century (Fig 5, yellow lines). A reduction in larger sized scallops influences both the socio-economics and the population dynamics of the fishery. A mismatch between population size distribution and selectivity of scallop dredges and targeting over time reduces potential landings in the absence of changes to management gear regulation or targeting. Scallop dredges are currently federally regulated to have 4-inch diameter rings in order to select specifically for scallops larger than 102mm [28]. Larger sized scallops also receive a price premium [18], so a reduction in the catch of U10 scallops (10 and under meats/lb) means that overall revenues are impacted when compared to lower OA impact scenarios. Biologically, scallop fecundity rates increase with age, size, and biomass, [32, 33]; thus, a population of larger scallops can lead to increased recruitment if populations are limited by larval supply (e.g. Mid-Atlantic Bight).

### 3.2 Impacts of management choices

**Fig 6:**
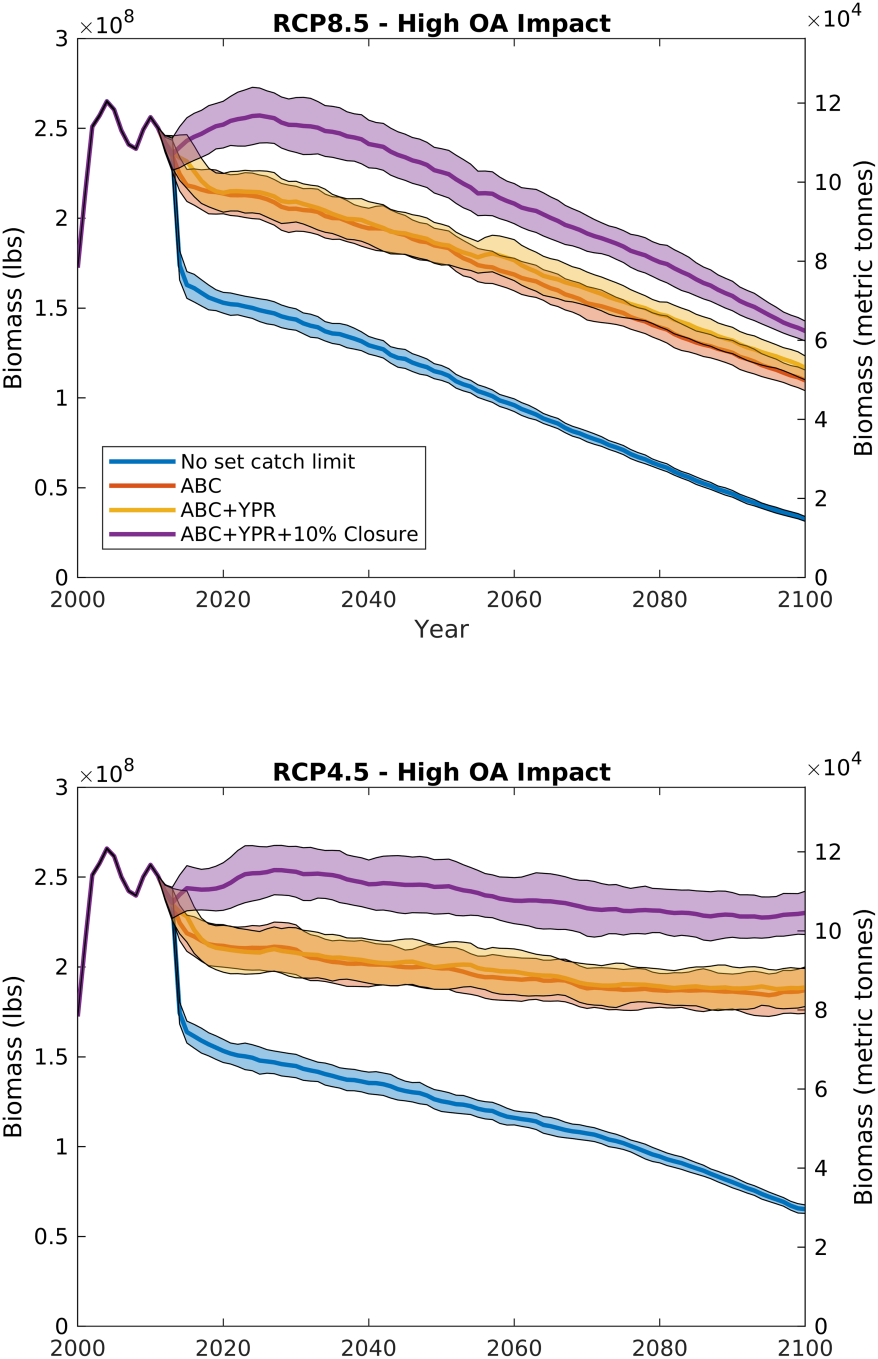
Scallop whole stock biomass from RCP8.5 (top panel) and RCP4.5 (bottom panel) under the highest ocean acidification impacts (increased larval mortality, reduced growth rates, and increased mortality due to predation) with varying management strategies: No set catch limit, maximum allowable biological catch (ABC), varying fisheries reference points (ABC+F_MSY_), and area closures (ABC+F_MSY_+10% closure). The purple line/band illustrates the same dataset as in Fig 3. Bold line illustrates the mean, and shaded area is the 95% confidence interval for 100 model runs with stochastic recruitment. The 2000–2012 period is hindcast with observed recruitment.

**Fig 7:**
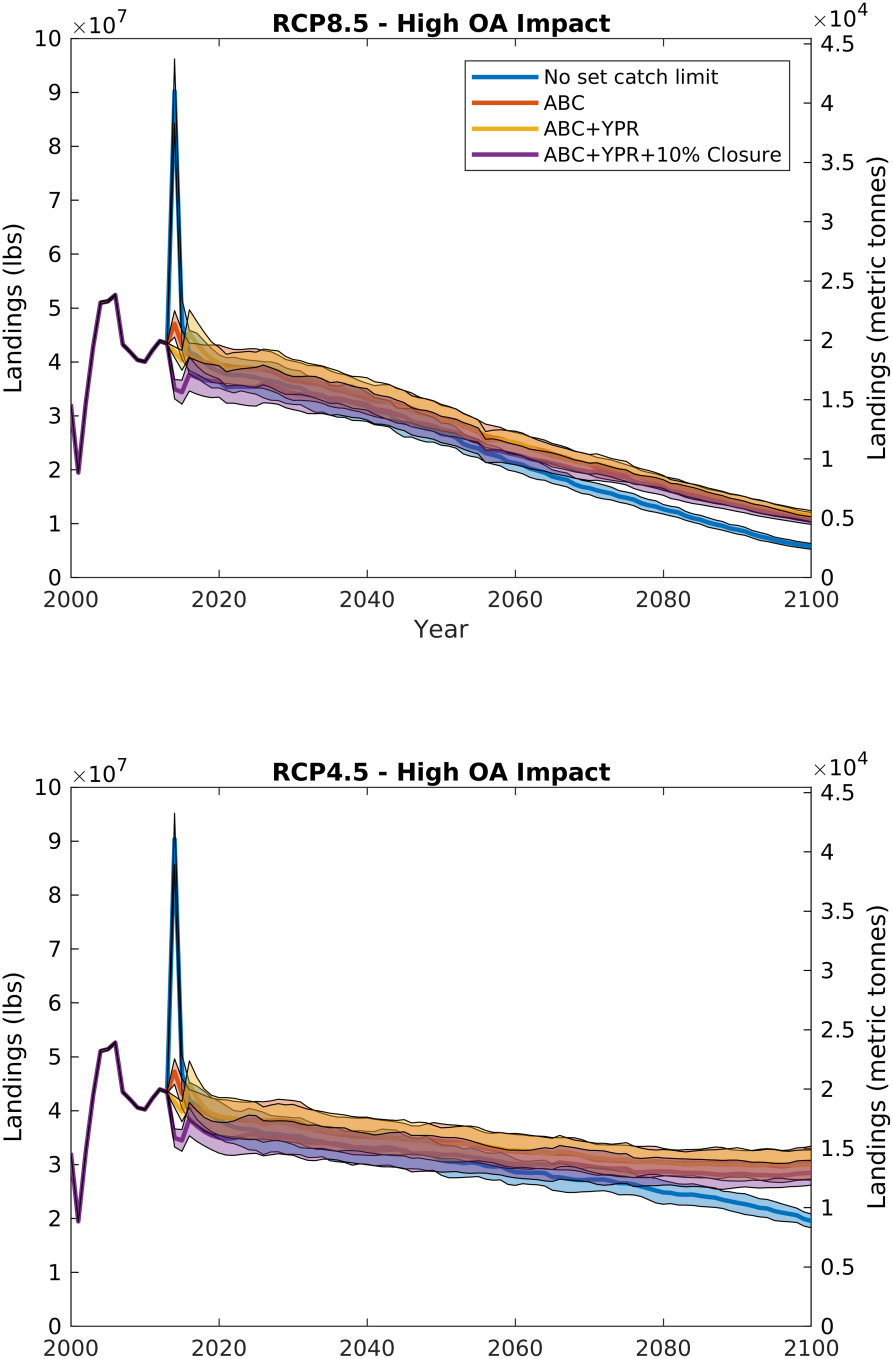
Scallop fishery landings from RCP8.5 (top panel) and RCP4.5 (bottom panel) under the highest ocean acidification impacts (increased larval mortality, reduced growth rates, and increased mortality due to predation) with management strategies: No set catch limit, maximum allowable biological catch (ABC), varying fisheries reference points (ABC+F_MSY_), and area closures (ABC+F_MSY_+10% closure). The purple line/band illustrates the same dataset as in Fig 4. Bold line illustrates the mean, and shaded area is the 95% confidence interval for 100 model runs with stochastic recruitment. The 2000–2012 period is hindcast with observed recruitment.

Management choice has by far the largest immediate impact on biomass and fishery outcomes. Although the “market chooses” scenario is unlikely to ever occur due to federal fisheries management regulations, the removal of a set catch limit is still an interesting exercise to highlight the value in the fisheries management process. Under the “no set catch limits” scenario, regardless of other possible impact pathways, there were dramatic declines in fishery biomass (Fig 6). For orientation, the red curves in Figs 3 and 6 correspond to the same cases (high management and high ocean acidification impact). Stock biomass was depleted by between ~30–80% for “no set catch limits” cases when compared to a similar climate and ocean acidification impact scenario that includes management set catch limits. Initially, when catch limits are removed, there is a spike in landings and decline in biomass suggesting that there are untapped resources within the sea scallop stock in any given year (blue data in Fig 7). Beyond this initial spike, landings do not deviate from the similar climate-with-high-management scenario (purple data in Fig 7) until beyond 2050. As landings are similar for the first 40 years of simulation, revenues also do not deviate between the “market chooses” and high management scenario until the latter half of the century. However, because stock biomass is depleted, scallop vessels must fish nearly twice as many days at sea to maintain landings at the same level, leading to significant reductions in crew incomes when compared to scenarios with set catch limits. Because operating costs of the industry are taken from crew shares [13, 18], after the initial spike, owner profitability remains largely similar between “market chooses” and management scenarios until landings and revenues deviate due to future climate impacts beyond 2050.

**Fig 8:**
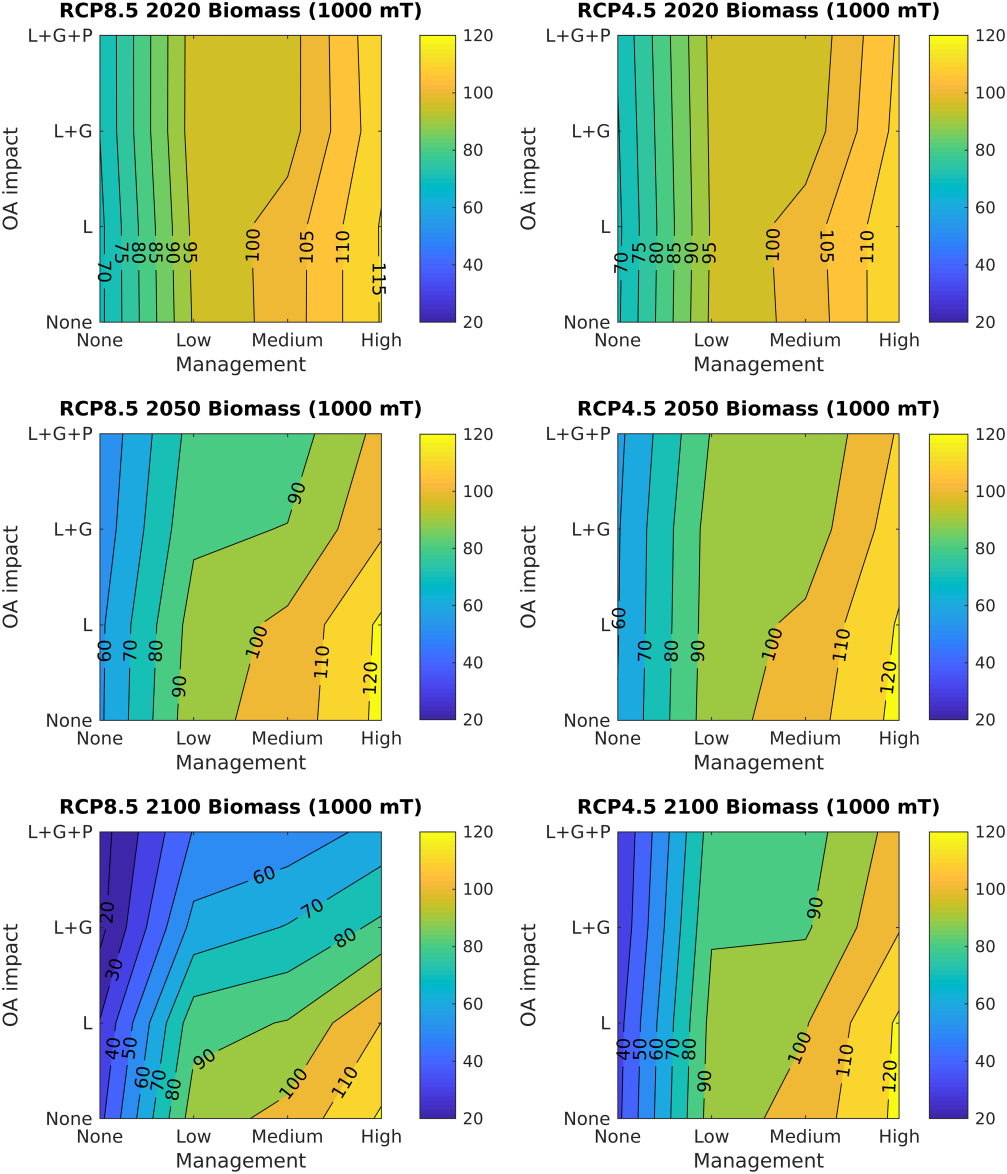
Contour plots of biomass at three different time points (2020, 2050, and 2100) for RCP8.5 (left panels) and RCP4.5 (right panels). X-axes increase management levels from low to high as no set catch limit (None), allowable biological catch limits only (low), ABC and variable fishing mortality at maximum sustainable yield (YPR, medium), and ABC, YPR, and an additional 10% closed area (high). Y-axes increase ocean acidification impacts from no impact to high impacts as no ocean acidification impacts, larval impacts only (L), larvae and growth rate impacts (L+G), and larvae, growth, and predation (L+G+P). Biomass is shown in units of 1000 metric tons (mT).

Each increase in management level improves the future outcome of the fishery for every climate scenario. The addition of a simple catch limit (ABC only management scenario) without time-evolving fisheries reference points or closed areas leads to the most relative gain in future biomass compared to the higher management scenarios (Figs 7 and 8). Time-evolving reference points (ABC+YPR, “medium” management) lead only to minor gains in biomass with high ocean acidification impacts, likely because the fishery is currently well-managed at a precautionary fishing mortality. Under RCP8.5 and high ocean acidification impacts, the highest level of management considered here increases the future biomass of the fishery compared to constant management targets and no closures. The fishery has slightly reduced landings due to a lower set catch limit from the initial 10% biomass closure beginning in 2012. However, the closure increases total biomass by more than 25% due to buildup of large scallops in the closed areas, and improves recruitment in the Mid-Atlantic region where a clear stock-recruit relationship has been observed [29].

Although the future projection of scallop biomass improves with the highest levels of management considered here, this additional management level only somewhat reduces the long-term overall decline in biomass (Figs 6, 7 and 8) and changes in size distribution (Fig 5) due to ocean acidification impacts. When a 10% closure is added to the scallop management regime, biomass builds up in the largest scallop size classes compared to scenarios with catch limits only (solid vs. dashed lines), but this buildup becomes less pronounced over time, e.g. 2020 (blue data in Fig 5) vs. 2100 (yellow data in Fig 5) and with increasing ocean acidification impacts. This suggests the 10% closure may not be enough to preserve scallop biomass under future changes in carbonate mineral saturation state.

### 3.3 Data and knowledge limitations

These projected model scenarios showing stock and landing declines with time under elevated CO_2_ and increasing ocean acidification are not without caveats. No studies have been published to date on the impacts of ocean acidification on *P*. *magellanicus*, and the IAM relies on experimental studies of other bivalves, including Pectinids, to estimate the relative impacts of ocean acidification on juvenile and adult scallop growth rates [13]. Since the initial meta-analysis used in Cooley et al. [13], several new experiments have been published within the scallop taxa. Lagos et al. [34] found that juvenile *Argopecten purpuratus* was sensitive to ocean acidification, with reduced growth rates in experimental individuals compared to those reared under present day conditions. However, Ramajo et al. [35] found the opposite result, with increased growth and calcification rates of *A*. *purpuratus* in response to ocean acidification. Gobler et al. [36] found that *Argopecten irradians* juveniles were sensitive to acidification, but this sensitivity manifested itself in reduced survival rates, rather than a reduction in growth rates. Older work by White et al. [10, 11] also found that larval *A*. *irradians* was sensitive to acidification that manifested in both reduced larval survival and calcification rates.

Given both the species-specific and within-species variability that is typically observed among different populations (e.g. [34] vs. [35]), generalizing how *P*. *magellanicus* may respond to ocean acidification using studies from other species may lead to errors in estimating the population and fishery trajectory under future climate scenarios. From the studies used in Cooley et al. [13], and the additional studies above, experiments have mostly been conducted on estuarine or shallow water species, where seasonal or diel cycling may expose bivalves to low carbonate mineral saturation state under naturally varying conditions. These organisms may be more resilient to ocean acidification than those found in more stable, deeper continental shelf environments, like *P*. *magellanicus*.

A few studies have been conducted on a similarly long-lived Pectinid, *Pecten maximus*, found in deep-water environments in the Northeast Atlantic. Experiments there have focused on the juvenile and larval life stages, and the swimming capability of adults, and they have shown mixed responses [12, 15, 21, 37]. Sanders et al. [37] found no impact of ocean acidification on juvenile *P*. *maximus* growth when food was not limiting. However, irrespective of treatment, growth as measured by changes in shell height was negligible over their 90-day study, suggesting a longer-term experiment might be needed to determine the effects ocean acidification on growth of long-lived species such as *P*. *maximus* or *P*. *magellanicus*. Larval *P*. *maximus* life stages are also sensitive to ocean acidification, with experimental declines in survival, shell height, and increases in deformity rates with decreasing carbonate mineral saturation state [12]. Currently, results from Andersen et al. [12] are used to link carbonate mineral saturation state to recruitment in the IAM [13]. Although Schalkhausser et al. [15] found the swimming behavior of *P*. *maximus* was sensitive to ocean acidification and reduced clapping force, a repeated experiment on a different subpopulation showed a different, yet still acidification-sensitive swimming response [21]. Populations sourced from Norway were more sensitive and exhibited reduced clapping force, while *P*. *maximus* individuals sourced from France were only sensitive to acidification when incubated under warm water conditions [15, 21]. The high variability in response within a single species suggests that studies on the response of *P*. *magellanicus* to ocean acidification are warranted to improve future projections of populations under future climate scenarios.

Even if ocean acidification impacts specific to *P*. *magellanicus* were known, individual scallop responses may be difficult to translate to population-level impacts. For example, although the population submodel in the IAM incorporates increased larval mortality through effects on the stock-recruit relationship, larval and juvenile stages (under two-year-old scallops) are not explicitly modeled. An additional pre-recruit model, such as that described in Punt et al. [38] for the Alaskan red king crab, that incorporates growth rates, larval and juvenile mortality, settlement indicators [39] and environmental change, would better describe how increased larval or juvenile mortality might impact population-level recruitment. Finally, biological adaptation or acclimatization over time to increased ocean acidification stress could occur [40]. Future iterations of this work could also incorporate an adaptation-type response within our growth, mortality, and predation rate projections [41], which is beyond the scope of the current study.

## 4 Conclusions

Integrated assessment models (IAMs) provide frameworks for bridging climate change and socioeconomic projections with other analyses of vulnerability, impacts, adaptation opportunities and mitigation possibilities. The wild-caught commercial US Atlantic sea scallop fishery (*P*. *magellanicus*) is currently valuable, well managed, and healthy, but is also potentially sensitive to increasing atmospheric CO_2_, ocean acidification, and ocean climate warming [5]. In previous work [13], we reported on preliminary sensitivity studies using a new sea scallop IAM that incorporates: scallop recruitment and size-structured scallop population dynamics; ocean CO_2_ uptake, biogeochemistry, and warming; and scallop fishery management rules and socio-economic drivers. Here we conduct a more comprehensive analysis of the scallop IAM exploring scenarios that vary atmospheric CO_2_ and climate change, ocean acidification impacts, fishery management, and fuel costs. For future external forcing over the 21^st^ century, we utilize representative concentration pathways (RCPs) that provide internally consistent projections of atmospheric CO_2_, ocean warming, population, economic development, and carbon tax. This framework and a generalized version of the IAM could be applied to other species with similar life history characteristics such as relatively sessile and size-structured population dynamics provided information was available on important model parameterizations such as growth and mortality rates and sensitivity to ocean acidification.

The suite of possible scenarios presented here are intended to provide initial ways to consider the impacts of differing degrees of climate change and ocean acidification on a highly valuable, wild-caught, federally regulated fishery. Impacts on the fishery were substantially smaller for a moderate climate policy scenario (RCP4.5) in comparison to a business-as-usual scenario (RCP8.5), showing the value of climate mitigation efforts. Ocean acidification may lead to shifts in the size structure of the scallop stock, with ensuing biological, fishery management and economic impacts (e.g. declining fishery revenue for smaller sized scallops). Model runs with increased management partially offset the decline in the scallop fishery, but do not fully protect the population from the long-term impacts of global change. The IAM simulations emphasize the benefits of sustained monitoring and adaptive management of the US scallop fishery to avoid the worst possible consequences of ocean acidification. Additionally, the development of this suite of model scenarios highlights the lack of empirical data on the potential impacts of ocean acidification on *P*. *magellanicus* at the organismal, population, and community level including effects from recruitment and growth to predator-prey interactions. A suite of laboratory studies identifying the response of various *P*. *magellanicus* life stages to ocean acidification is a clear next step in determining the future of this valuable fishery.
